# The role of *FOXO3* polymorphisms in susceptibility to tuberculosis in a Chinese population

**DOI:** 10.1002/mgg3.770

**Published:** 2019-06-26

**Authors:** Bo Wang, Yuhe Wang, Li Wang, Xue He, Yongjun He, Mei Bai, Linhao Zhu, Jianwen Zheng, Dongya Yuan, Tianbo Jin

**Affiliations:** ^1^ Department of the 4th Internal Medicine Xi’an Chest Hospital Xi’an Shaanxi China; ^2^ Key Laboratory of Molecular Mechanism and Intervention Research for Plateau Diseases of Tibet Autonomous Region, School of Medicine Xizang Minzu University Xianyang Shaanxi China; ^3^ Department of Clinical Laboratory Affiliated Hospital of Xizang Minzu University Xianyang Shaanxi China; ^4^ School of Basic Medical Sciences Xizang Minzu University Xianyang Shaanxi China; ^5^ Department of Neurology Affiliated hospital of Xizang Minzu University Xianyang Shaanxi China; ^6^ Key Laboratory of Resource Biology and Biotechnology in Western China (Northwest University) Ministry of Education Xi’an Shaanxi China

**Keywords:** *FOXO3*, infectious diseases, *Mycobacteria tuberculosis*, polymorphism

## Abstract

**Background:**

Tuberculosis (TB) is a significant worldwide health problem, and is caused by *Mycobacteria tuberculosis*. Recent studies have suggested that *FOXO3* plays vital roles in the risk of immune–related infectious diseases such as TB.

**Methods and Results:**

The present study aimed to evaluate *FOXO3* genetic variants and TB risk. We recruited 510 TB patients and 508 healthy controls in this study. All subjects were genotyped with the Agena MassARRAY platform. Odds ratios (ORs) and 95% confidence intervals (CIs) were calculated using logistic regression adjusted for age and gender. Our result revealed that rs3800229 T/G and rs4946935 G/A genotypes significantly increased the risk of TB (OR = 1.34, 95% CI = 1.04–1.74, *p* = 0.026; OR = 1.34, 95% CI = 1.03–1.73, *p* = 0.029, respectively). In stratified analysis according to gender and age, we observed that rs3800229 T/G and rs4946935 G/A genotypes were associated with an increase the risk of TB among males and age ≤41 years, respectively (OR = 1.47, 95% CI = 1.06–2.04, *p* = 0.022 and OR = 1.45, 95% CI = 1.05–2.02, *p* = 0.025).

**Conclusions:**

Our study showed that rs3800229 and rs4946935 in *FOXO3* were associated with a risk of TB in the Chinese population.

## 1 INTRODUCTION

Tuberculosis (TB), as one of the most common chronic infectious disease, is caused by *Mycobacteria tuberculosis* and remains high morbidity and mortality in our world. Among those infected with *Mycobacterium tuberculosis*, approximately 5%–15% develop clinical disease during their lifetime (Chen, Liu, Zhu, Yang, & Lu, 2013). It is reported that there are about 10.0 million new TB cases and 1.3 million deaths occurred in 2017 (WHO. Global tuberculosis report 2018). Studies suggest that host genetic variants play a crucial role in determining resistance or susceptibility to TB disease (Liu et al., 2015; Rodriguez‐Castillo et al., 2015). However, the molecular mechanism of the genetic predisposition to TB is still largely unknown.

Recently, several candidate TB–susceptibility genes have been identified, including Vitamin D receptor gene (Sun et al., 2015), *CYP7A1* (Qrafli et al., 2014), *CD14* (Miao, Ge, Xu, & Xu, 2014), *TLR9* (Bharti et al., 2014) and *SEC14L2* (Du et al., 2017). *FOXO3* belongs to the Forkhead family of transcription factors, primarily identified as downstream targets of insulin/IGF–1 signaling pathway (Hosaka et al., 2004). *FOXO3* is known to play a role in modulating immune response and in response to growth factors (Hedrick, Hess Michelini, Doedens, Goldrath, & Stone, 2012). Several SNPs were found to be associated with increased lifespan in the German and Han Chinese population (Flachsbart et al., 2017; Sun et al., 2015). In addition, in the immune system, it has been reported that *FOXO3* is an important regulator of T cell‐，dendritic cell‐ and macrophage‐ homeostasis (Dejean et al., 2009; Watkins & Hurwitz, 2012). Previous genome–wide association studies have shown that *FOXO3* gene variants were observed to be associated with severity of autoimmune diseases and infectious diseases, such as Crohn's disease and malaria (Lee et al., 2013; Marlow, Han, Triggs, & Ferguson, 2015). However, there were few reports regarding the impact of *FOXO3* genetic variants polymorphism on susceptibility to TB (Lu et al., 2016).

Thus, in this study, our primary objective was to evaluate the association between *FOXO3* genetic variants and the risk of TB in a sample of Chinese population.

## 2 MATERIALS AND METHODS

### 2.1 Study populations

In this study, we recruited 510 TB patients and 508 age– and sex–matched healthy controls from Xi'an Chest Hospital, Shaanxi Province. All TB patients and healthy controls were unrelated to the Han Chinese population. Patients were diagnosed by specialized doctors according to clinical signs, chest x–ray examination and positive sputum smear for acid–fast bacilli. All healthy controls underwent an x–ray examination, none of them showed any clinical characteristics of TB. TB patients and healthy controls, who were HIV positive, autoimmune diseases, chronic inflammatory or other diseases affecting host immunity were excluded. Blood samples were obtained from each subject for molecular analysis.

### 2.2 Genotyping

Genomic DNA was extracted from whole blood samples with GoldMag whole blood genomic DNA purification kit (GoldMag Co. Ltd. Xi'an city, China). DNA concentration was evaluated with a NanoDrop 2000 platform (Thermo Fisher S 95% CIentific, Waltham, MA, USA), according to the manufacturer's instruction. The genes associated with TB were selected from the Genomic Wide Association Study using UCSC (http://genome.ucsc.edu/) database. We found that *FOXO3* was associated with several diseases including TB (Greer & Brunet, 2008). The candidate SNPs of *FOXO3* were selected from the 1,000 Genomes Project data (http://www.internationalgenome.org/) with minor allele frequency (MAF) > 0.02 in the Asian population and MAF > 0.05 in the Chinese Han Beijing population. Finally, four SNPs in *FOXO3* were selected in this case–control study. The Agena Bioscience Assay Design Suite V2.0 software (https://agenacx.com/online–tools/) was performed to analyze the amplification and extension primers. The MassARRAY Nanodispenser and MassARRAY iPLEX platform (both from Agena Bios 95% CIence, San Diego, CA, USA) were used to genotype the SNPs. Data management was performed using Agena Bioscience TYPER version 4.0 software as previously described (Zhu et al., 2017).

### 2.3 Statistical analysis

The base characteristics such as age and gender of TB patients and healthy controls were conducted using the SPSS 20.0 (SPSS Inc., Chicago, IL, USA) software. Further, the following analysis was performed through PLINK software (version 1.07). Deviation from Hardy–Weinberg equilibrium (HWE) was assessed using the Chi–square test to compare the observed and expected genotype frequencies among the control subjects. Allele and genotype frequencies were compared between TB patients and healthy controls using the χ^2^ test and Fisher's exact test. To evaluate the association between *FOXO3* SNPs and the risk of TB, we calculated odds ratios (ORs) and 95% confidence intervals (CIs) adjustment gender and age. We also applied four different genetic models: the codominant model, the dominant model, the recessive model and the log–additive model to assess *FOXO3* SNPs associations with TB risk using PLINK software (version 1.07). Power and Sample Size Calculation software (http://biostat.mc.vanderbilt.edu/wiki/Main/PowerSampleSize) were used to calculate the power of the significant difference. The haplotypes of the candidate SNPs were analyzed using Haploview v4.2. Linkage disequilibrium (LD) between SNPs was analyzed using the pairwise LD measure D′. Pairwise D′ > 0.80 suggested that the SNPs were in high LD. For all statistical tests, *p* < 0.05 was considered statistically significant.

## 3 RESULTS

A total of 1,018 individuals including 510 TB patients (mean age, 41.90 ± 14.83 years old; 318 males and 192 females) and 508 healthy controls (mean age, 41.14 ± 18.42 years old; 316 males and 192 females) were recruited in this study. There was no significant difference among the groups regarding age (*p* = 0.469) and gender (*p* = 0.961). The general demographic characteristics are shown in Table 1.

**Table 1:** Demographic distribution of TB patients and Healthy controls

Variants	TB (*n* = 510)	Controls (*n* = 508)	*p*
Age, year (mean ± *SD*)	41.90 ± 14.83	41.14 ± 18.42	0.469
Gender			0.961
Male	318 (62.35%)	316 (62.20%)	
Female	192 (37.65%)	192 (37.80%)	

Note

*p* values were calculated using χ^2^ test/Fisher's exact test.

Abbreviation:* SD*: standard deviation.

*P* < 0.05 indicates statistical significance.

Detailed SNPs information including the position, MAF and HWE *p* value are listed in Table 2. The MAF of all SNPs were more than 5% in our study population. All candidate SNPs accorded with HWE (*p* > 0.05).

**Table 2:** Basic characteristics and allele frequencies among *FOXO3* SNPs

SNP	Chr	Allele	MAF		HWE *p*–Value
			Case	Control	
rs1536057	6	A/G	0.18	0.16	0.411
rs12212067	6	G/T	0.13	0.12	0.290
rs3800229	6	G/T	0.24	0.22	0.249
rs4946935	6	A/G	0.24	0.22	0.307

Note

Sites with HWE, *p* < 0.05, are excluded.

*p* values calculated with two–sided χ^2^.

Abbreviations: CI, confidence interval; HWE, Hardy–Weinberg equilibrium; MAF, minor allele frequency, OR, odds ratio; SNP, single nucleotide polymorphism.

Differences in SNPs genotype and allele frequencies distribution between TB patients and healthy controls were analyzed using Chi‐squared test and ORs to assess the associations with TB risk, as displayed in Table 3. The minor allele of each SNP as a risk factor was compared to the wild‐type allele. However, we had not observed that any SNPs were significantly different of allele frequencies between cases and healthy controls (Table 3). Further, different genetic models were performed to evaluate the potential association between the *FOXO3* polymorphism and the risk of TB after adjusting age and gender. We observed that rs380029 T/G genotype was associated with increased the risk of TB in comparison to the common genotype TT (OR = 1.34, 95% CI = 1.04–1.74, *p* = 0.026), with power values of 0.639. Similarly, compared with the common genotype G/G, the genotype G/A of rs4946935 was significantly associated with TB risk (OR = 1.34, 95% CI = 1.03–1.73, *p* = 0.029, power = 0.639). However, no significant correlation was identified between rs380029 and rs4946935 genotypes and TB risk in the dominant model, recessive model and log–additive model.

**Table 3:** The association between four SNPs within *FOXO3* and the risk of tuberculosis

SNP	Model	Genotype	TB (*n* %)	Controls (*n* %)	Adjusted by gender and age		Crude analysis	
					OR (95% CI)	*p*	OR (95% CI)	*p*
rs1536057	Allele	G	838 (82.16)	852 (83.86)	Baseline			
		A	182 (17.84)	164 (16.14)	1.13 (0.90–1.42)	0.307		
	Codominant	G/G	340 (66.67)	360 (70.87)	Baseline		Baseline	
		G/A	158 (30.98)	132 (25.98)	1.28 (0.97–1.68)	0.083	1.27 (0.96–1.67)	0.091
		A/A	12 (2.35)	16 (3.15)	0.79 (0.37–1.69)	0.538	0.79 (0.37–1.70)	0.554
	Dominant	G/G	340 (66.67)	360 (70.87)	Baseline		Baseline	
		G/A—A/A	170 (33.33)	148 (29.13)	1.22 (0.94–1.59)	0.140	1.22 (0.93–1.59)	0.149
	Recessive	G/G—G/A	498 (97.65)	492 (96.85)	Baseline		Baseline	
		A/A	12 (2.35)	16 (3.15)	0.73 (0.34–1.57)	0.424	0.74 (0.35–1.58)	0.439
	Log–additive	–	–	–	1.13 (0.90–1.43)	0.295	1.13 (0.90–1.43)	0.305
rs12212067	Allele	T	887 (86.96)	894 (87.99)	Baseline			
		G	133 (13.04)	122 (12.01)	1.10 (0.84–1.43)	0.482		
	Codominant	T/T	385 (75.49)	396 (77.95)	Baseline		Baseline	
		T/G	117 (22.94)	102 (20.08)	1.19 (0.88–1.61)	0.261	1.18 (0.87–1.59)	0.280
		G/G	8 (1.57)	10 (1.97)	0.82 (0.32–2.09)	0.673	0.82 (0.32–2.11)	0.684
	Dominant	T/T	385 (75.49)	360 (77.95)	Baseline		Baseline	
		T/G—G/G	125 (24.51)	148 (22.05)	1.16 (0.86–1.55)	0.334	1.15 (0.86–1.54)	0.353
	Recessive	T/T –T/G	502 (98.43)	498 (98.03)	Baseline		Baseline	
		G/G	8 (1.57)	10 (1.97)	0.79 (0.31–2.01)	0.617	0.79 (0.31–2.03)	0.629
	Log–additive	–	–	–	1.10 (0.85–1.43)	0.469	1.10 (0.85–1.42)	0.486
rs3800229	Allele	T	775 (75.98)	789 (77.66)	Baseline			
		G	245 (24.02)	227 (22.34)	1.10 (0.89–1.35)	0.370		
	Codominant	T/T	285 (55.88)	311 (61.22)	Baseline		Baseline	
		T/G	205 (40.20)	167 (32.87)	1.34 (1.04–1.74)	0.026*	1.34 (1.03–1.74)	0.028*
		G/G	20 (3.92)	30 (5.91)	0.73 (0.41–1.31)	0.294	0.73 (0.40–1.31)	0.289
	Dominant	T/T	285 (55.88)	311 (61.22)	Baseline		Baseline	
		T/G—G/G	225 (44.12)	197 (38.78)	1.25 (0.97–1.60)	0.081	1.25 (0.97–1.6)	0.084
	Recessive	T/T –T/G	490 (96.08)	478 (94.09)	Baseline		Baseline	
		G/G	20 (3.92)	30 (5.91)	0.65 (0.37–1.17)	0.149	0.65 (0.36–1.16)	0.146
	Log–additive	–	–	–	1.10 (0.90–1.36)	0.355	1.10 (0.89–1.36)	0.364
rs4946935	Allele	G	775 (75.98)	790 (77.76)	Baseline			
		A	245 (24.02)	226 (24.24)	1.11 (0.90–1.36)	0.342		
	Codominant	G/G	285 (55.88)	311 (61.22)	Baseline		Baseline	
		G/A	205 (40.20)	168 (33.07)	1.34 (1.03–1.73)	0.029*	1.33 (1.03–1.73)	0.031*
		A/A	20 (3.92)	29 (5.71)	0.75 (0.42–1.36)	0.351	0.75 (0.42–1.36)	0.347
	Dominant	G/G	285 (55.88)	311 (61.22)	Baseline		Baseline	
		G/A—A/A	225 (44.12)	197 (38.78)	1.25 (0.97–1.60)	0.081	1.25 (0.97–1.60)	0.084
	Recessive	G/G—G/A	490 (96.08)	479 (94.29)	Baseline		Baseline	
		A/A	20 (3.92)	29 (5.71)	0.68 (0.38–1.21)	0.188	0.67 (0.38–1.21)	0.185
	Log–additive	–	–	–	1.11 (0.90–1.37)	0.327	1.11 (0.90–1.37)	0.335

Abbreviations: CI, confidence interval; OR, odds ratio; SNP: single nucleotide polymorphism.

* *p* < 0.05 indicates statistical significance.

Moreover, in accordance with the stratified analyses by gender and age. We found that the T/G genotype of rs3800229 and the G/A genotype of rs4946935 were associated with increased risk of TB in males (OR = 1.47, 95% CI = 1.06–2.04, *p* = 0.022; OR = 1.45, 95% CI = 1.05–2.02, *p* = 0.025, respectively) with power values of 0.825 and 0.798, respectively. There was no significant association between rs380029 and rs4946935 genotypes and TB risk in females (Table 4). Besides, the distribution frequency of rs3800229 T/G and T/G—G/G genotype were higher in TB risk with age ≤41 years (OR = 1.55, 95% CI = 1.07–2.26, *p* = 0.020, power = 0.929; OR = 1.44, 95% CI = 1.01–2.06, *p* = 0.044, power = 0.817, respectively). Our results also suggested that rs4946935 G/A and G/A—A/A genotypes were significantly associated with TB risk with age ≤41 years (OR = 1.55, 95% CI = 1.07–2.26, *p* = 0.020, power = 0.929 and OR = 1.44, 95% CI = 1.01–2.06, *p* = 0.044, power = 0.817, respectively). No association of rs3800229 and rs4946935 genotype frequencies and TB risk were observed  with age > 41 years. (Table 5).

**Table 4:** The association between two SNPs within *FOXO3* and the risk of tuberculosis stratified by gender

SNP	Model	Genotype	Male		Female	
			OR (95% CI)	*p^a^*	OR (95% CI)	*p* ^*a*^
rs3800229	Codominant	T/T	Baseline		Baseline	
		T/G	1.47 (1.06–2.04)	0.022*	1.14 (0.74–1.75)	0.563
		G/G	0.54 (0.24–1.22)	0.139	1.06 (0.44–2.54)	0.896
	Dominant	T/T	Baseline		Baseline	
		T/G—G/G	1.33 (0.97–1.82)	0.081	1.12 (0.75–1.69)	0.576
	Recessive	T/T –T/G	Baseline		Baseline	
		G/G	0.46 (0.21–1.04)	0.062	1.01 (0.43–2.40)	0.976
	Log–additive	–	1.12 (0.85–0.85)	0.417	1.08 (0.78–1.51)	0.641
rs4946935	Codominant	G/G	Baseline		Baseline	
		G/A	1.45 (1.05–2.02)	0.025*	1.14 (0.74–1.75)	0.563
		A/A	0.57 (0.25–1.30)	0.179	1.06 (0.44–2.54)	0.896
	Dominant	G/G	Baseline		Baseline	
		G/A—A/A	1.33 (0.97–1.82)	0.081	1.12 (0.75–1.69)	0.576
	Recessive	G/G—G/A	Baseline		Baseline	
		A/A	0.49 (0.22–1.11)	0.086	1.01 (0.43–2.40)	0.976
	Log–additive	–	1.13 (0.86–0.86)	0.378	1.08 (0.78–1.51)	0.641

Abbreviations: CI, confidence interval; OR, odds ratio; SNP: single nucleotide polymorphism.

* *p^a^* < 0.05 indicates statistical significance.

**Table 5:** The association between two SNPs within *FOXO3* and the risk of tuberculosis stratified by age

SNP	Model	Genotype	≤41		>41	
			OR (95% CI)	*p^a^*	OR (95% CI)	*p^a^*
rs3800229	Codominant	T/T	Baseline		Baseline	
		T/G	1.55 (1.07–2.26)	0.020*	1.05 (0.71–1.57)	0.793
		G/G	0.89 (0.40–1.97)	0.769	0.62 (0.24–1.62)	0.331
	Dominant	T/T	Baseline		Baseline	
		T/G—G/G	1.44 (1.01–2.06)	0.044*	1.00 (0.68–1.46)	0.994
	Recessive	T/T –T/G	Baseline		Baseline	
		G/G	0.75 (0.34–1.64)	0.470	0.61 (0.24–1.56)	0.305
	Log–additive	–	1.23 (0.92–1.65)	0.165	0.67 (0.26–1.76)	0.420
rs4946935	Codominant	G/G	Baseline		Baseline	
		G/A	1.55 (1.07–2.26)	0.020*	1.04 (0.70–1.54)	0.843
		A/A	0.89 (0.40–1.97)	0.769	0.68 (0.26–1.80)	0.444
	Dominant	G/G	Baseline		Baseline	
		G/A—A/A	1.44 (1.01–2.06)	0.044*	1.00 (0.68–1.46)	0.994
	Recessive	G/G—G/A	Baseline		Baseline	
		A/A	0.75 (0.34–1.64)	0.470	0.67 (0.26–1.76)	0.420
	Log–additive	–	1.23 (0.92–1.65)	0.165	0.94 (0.68–1.30)	0.712

Abbreviations: CI, confidence interval; OR, odds ratio; SNP: single nucleotide polymorphism.

* *p^a^* < 0.05 indicates statistical significance.

**Figure 1:**
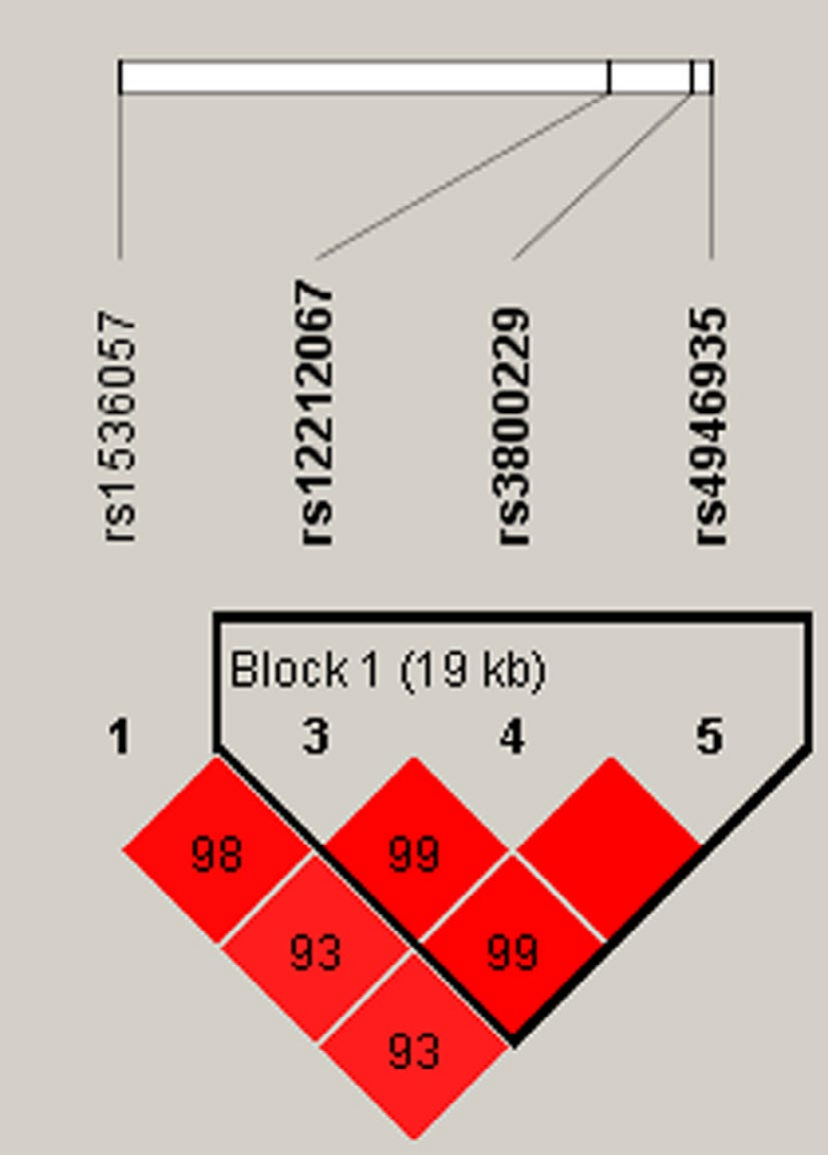
Haplotype block map for SNPs in *FOXO3* . The numbers inside the diamonds indicate the D′ for pairwise analyses

Subsequently, the association of *FOXO3* with TB susceptibility was explored through haplotype analysis. LD analysis confirmed that SNPs rs12212067, rs3800229 and rs4946935 compose an LD block, as shown in Figure 1. Pairwise D′ value between SNPs rs12212067 and rs3800229 was 0.99. Pairwise D′ value between SNPs rs12212067 and rs4946935 was 0.99. Pairwise D′ value between SNPs rs3800229 and rs4946935 was 1.00. The results indicated that these SNPs were high in LD (all pairwise D′ > 0.80). To examine the effect of haplotype on the risk of TB, the haplotype‐based logical regression method adjusted by age and gender was performed. However, our results did not reveal a significant correlation between common haplotype and TB risk (all *p* > 0.05). The result is presented in Table 6.

**Table 6:** Haplotype analysis of *FOXO3* SNPs in association with the risk of tuberculosis

Haplotype	Freq		*p*–Value	Crude analysis		Adjusted by gender and age	
	TB	Controls		OR (95% CI)	*p*	OR (95% CI)	*p*
GGA	0.13	0.12	0.524	1.09 (0.84–1.41)	0.526	1.09 (0.84–1.42)	0.508
TGA	0.89	0.90	0.538	0.92 (0.69–1.21)	0.539	0.92 (0.69–1.22)	0.545
TTG	0.76	0.78	0.343	0.90 (0.73–1.11)	0.338	0.90 (0.73–1.11)	0.329

Note

Block comprised of the three closely linked SNPs rs12212067, rs3800229 and rs4946935.

*p* values were calculated with χ^2^ tests.

Abbreviations: CI, confidence interval; OR, odds ratio.

*p* < 0.05 indicates statistical significance.

## 4 DISCUSSION

Previous studies confirmed that host genetic variants play an important role in TB infection and progress, and several candidate genes have been identified (Du et al., 2017; Qrafli et al., 2014; Sun et al., 2015). However, the association between *FOXO3* variations polymorphism and TB risk is poorly understood. In this study, we investigated the relationship of *FOXO3* variants polymorphism and TB risk. The result suggested that the T to G mutation of rs3800229 and the G to A mutation of rs4946935 significantly increased the risk of TB in the Chinese population.

FOXO transcription factor is a subfamily of the FOX family, which consists of four members including *FOXO1*, *FOXO3*, *FOXO4* and *FOXO6* (Hosaka et al., 2004). These transcription factors have a wide range of functions, such as cellular differentiation, cell–cycle control, oxidative stress, apoptosis and other cellular functions (Greer & Brunet, 2008; Maiese, Chong, Hou, & Shang, 2009). *FOXO3* is 124.94 kb in length and located on chromosome 6q21. Increasing evidence shows that *FOXO3* plays an important role in the human immune system (Nguetse, Kremsner, & Velavan, 2015). It is involved in the regulation of several immune functions in dendritic and T cells as well as in macrophages (Dejean et al., 2009; Watkins & Hurwitz, 2012).

Recently, the studies confirmed that *FOXO3* contributed to the pathogenesis of several immune associated disorder, including TB (Haoues et al., 2014; Lu et al., 2016). In the study of Lee et al, it was suggested that the minor G allele of rs12212067 in *FOXO3* is associated with a milder course of Crohn's disease, rheumatoid arthritis and with increased risk of severe malaria, which through down–regulation of proinflammatory cytokines including *TNFα* and up–regulation of anti–inflammatory cytokines including *IL–10*. (Junqueira et al., 2010; Langhorne et al., 2004; Lee et al., 2013). Sun et al revealed that the G allele of rs12212067 in *FOXO3* might effected *FOXO3* expression, subsequently affecting the TB progress in active TB patients (Lu et al., 2016). These researches indicate that maintenance of suitable *FOXO3* levels is critical in preventing TB. The mutation of *FOXO3* polymorphisms leads to change in the activity of the transcription factor and increases susceptibility for TB.

As we all know, such studies have confirmed that age and gender play a crucial role in the etiology of TB (Zhang et al., 2011). The social, environment and lifestyle factors have also been found to be associated with TB risk in the world (Murray, Oxlade, & Lin, 2011; Wang & Shen, 2009). Thus, persons may have different association with TB risk in different gender and age. In this study, our stratified analyses discover a significant effect of rs3800229 T/G and rs4946935 G/A genotypes on risk of TB among males and age ≤41 years, respectively.

Examination of haplotype frequencies and LD patterns was also important for identification of the appropriated populations for tag SNP selection (Gabriel et al., 2002). Our results suggested that rs12212067, rs3800229 and rs4946935 compose an LD block (all pairwise D′ > 0.80). The results did not reveal a significant correlation between common haplotype and TB risk (all *p* > 0.05).

Furthermore, Regulome DB (http://www.regulomedb.org/) and the prediction tools SNPinfo (https://snpinfo.niehs.nih.gov/snpinfo/snpfunc.html) were performed to determine the potential functional effect of the SNPs. No data were provided for the functional prediction of rs3800229 and rs4946935. Hence, further molecular characterization is need to explore in vivo and in vitro experiments.

There were several limitations in our study. First, in the case–control study, the selection bias could not be avoided due to the hospital‐based design. Second, the information of our samples were incomplete, the association between clinical parameters and TB risk were need to explore. In the future, we would like to collect clinical characteristics to evaluate this difference between the case and control group.

In conclusion, our result is the first to confirm that rs3800229 and rs4946935 of *FOXO3* were associated with increased risk of TB in a Han Chinese population. In further studies, the causal variant(s) and the molecular mechanisms underlying the association with TB need to be researched.

## CONFLICTS OF INTEREST

The authors have declared that they have no conflict of interest.

## ETHICS APPROVAL

All participants were informed in writing and verbally of the procedures, and purpose of this study and signed informed consent documents were obtained from both patients and controls. Study protocols were approved by the ethics committee of the Xi'an Chest Hospital, and complied with the ethical standards of the Ethical Committee and World Medical Association Declaration of Helsinki. All research analyses were carried out in accordance with the approved guidelines and regulations.
